# Age dependent associations of risk factors with heart failure: pooled population based cohort study

**DOI:** 10.1136/bmj.n461

**Published:** 2021-03-23

**Authors:** Jasper Tromp, Samantha M A Paniagua, Emily S Lau, Norrina B Allen, Michael J Blaha, Ron T Gansevoort, Hans L Hillege, Douglas E Lee, Daniel Levy, Ramachandran S Vasan, Pim van der Harst, Wiek H van Gilst, Martin G Larson, Sanjiv J Shah, Rudolf A de Boer, Carolyn S P Lam, Jennifer E Ho

**Affiliations:** 1 National Heart Centre Singapore, Singapore; 2 Duke-NUS Medical School, Singapore; 3 Department of Cardiology, University of Groningen, University Medical Centre Groningen, Groningen, Netherlands; 4 Cardiovascular Research Center, Department of Medicine, Massachusetts General Hospital, Boston, MA, USA; 5 Corrigan-Minehan Heart Center, Cardiology Division, Department of Medicine, Massachusetts General Hospital, Boston, MA, USA; 6 Department of Epidemiology, Feinberg School of Medicine, Northwestern University, Chicago, IL, USA; 7 Ciccarone Center for the Prevention of Heart Disease, Johns Hopkins University, Baltimore, MD, USA; 8 Department of Internal Medicine, University of Groningen, University Medical Centre Groningen, Groningen, Netherlands; 9 Institute for Clinical Evaluative Sciences, Toronto, Canada; 10 Framingham Heart Study, Framingham, MA, USA; 11 Center for Population Studies of the National Heart, Lung, and Blood Institute, Bethesda, MD, USA; 12 Cardiovascular Medicine Section, Department of Medicine and Section of Preventive Medicine and Epidemiology, Boston University School of Medicine, Boston, MA, USA; 13 Department of Mathematics and Statistics, Boston University, Boston, MA, USA; 14 Division of Cardiology, Northwestern University Feinberg School of Medicine, Chicago, IL, USA; * Contributed equally

## Abstract

**Objective:**

To assess age differences in risk factors for incident heart failure in the general population.

**Design:**

Pooled population based cohort study.

**Setting:**

Framingham Heart Study, Prevention of Renal and Vascular End-stage Disease Study, and Multi-Ethnic Study of Atherosclerosis.

**Participants:**

24 675 participants without a history of heart failure stratified by age into young (<55 years; n=11 599), middle aged (55-64 years; n=5587), old (65-74 years; n=5190), and elderly (≥75 years; n=2299) individuals.

**Main outcome measure:**

Incident heart failure.

**Results:**

Over a median follow-up of 12.7 years, 138/11 599 (1%), 293/5587 (5%), 538/5190 (10%), and 412/2299 (18%) of young, middle aged, old, and elderly participants, respectively, developed heart failure. In young participants, 32% (n=44) of heart failure cases were classified as heart failure with preserved ejection fraction compared with 43% (n=179) in elderly participants. Risk factors including hypertension, diabetes, current smoking history, and previous myocardial infarction conferred greater relative risk in younger compared with older participants (P for interaction <0.05 for all). For example, hypertension was associated with a threefold increase in risk of future heart failure in young participants (hazard ratio 3.02, 95% confidence interval 2.10 to 4.34; P<0.001) compared with a 1.4-fold risk in elderly participants (1.43, 1.13 to 1.81; P=0.003). The absolute risk for developing heart failure was lower in younger than in older participants with and without risk factors. Importantly, known risk factors explained a greater proportion of overall population attributable risk for heart failure in young participants (75% *v* 53% in elderly participants), with better model performance (C index 0.79 *v* 0.64). Similarly, the population attributable risks of obesity (21% *v* 13%), hypertension (35% *v* 23%), diabetes (14% *v* 7%), and current smoking (32% *v* 1%) were higher in young compared with elderly participants.

**Conclusions:**

Despite a lower incidence and absolute risk of heart failure among younger compared with older people, the stronger association and greater attributable risk of modifiable risk factors among young participants highlight the importance of preventive efforts across the adult life course.

## Introduction

Heart failure affects up to one in five adults during their lifetime and is associated with considerable mortality and morbidity, despite advances in management.^1^ ^2^ ^3^ Although most of the burden of heart failure is borne by people aged 65 years or over, recent reports from Denmark and Sweden show an increase in the incidence of heart failure particularly among younger people.^4^ ^5^ ^6^ Data from the Swedish conscript registry suggest that an increase in the prevalence of risk factors for heart failure, particularly obesity, at a young age is responsible for this increasing burden of heart failure early in life.^4^ This is in line with several reports on patients with prevalent heart failure, whereby younger patients with heart failure (both with a reduced pumping function (heart failure with reduced ejection fraction) and a reduced filling function (heart failure with preserved ejection fraction)) were more often non-white, obese men with diabetes, with a relatively lower prevalence of other non-cardiac comorbidities.^7^ ^8^ ^9^ ^10^ ^11^ Taken together, these findings suggest that important age differences in clinical phenotypes might exist among patients with heart failure.

In this context, we sought to examine whether age modified the effect of traditional risk factors on the development of future heart failure. Specifically, we sought to identify the age dependent incidence of new onset heart failure, heart failure with reduced ejection fraction, and heart failure with preserved ejection fraction; investigate the differential association of risk factors with new onset heart failure; and study the relative contribution of risk factors to the incidence of heart failure according to age strata. Understanding age dependent differences in risk factors leading up to development of heart failure may shed light on observed differences in clinical phenotypes across age and ultimately may inform future preventive strategies.

## Methods

### Study sample

We included three observational, prospective, community based cohorts with adjudicated heart failure outcomes: the Framingham Heart Study (FHS) original and offspring cohorts, the Prevention of Renal and Vascular Endstage Disease (PREVEND) study, and the Multi-Ethnic Study of Atherosclerosis (MESA).^12^ ^13^ ^14^ ^15^ We included participants from the following baseline examinations: FHS original cohort examination 16 (1979-82) and 24 (1995-98), FHS offspring cohort examination 2 (1979-83) and 6 (1995-98), PREVEND examination 1 (1997-98), and MESA examination 1 (2000-02). We excluded participants with prevalent heart failure (n=195), missing follow-up (n=27), or missing covariates (n=1063), resulting in a total sample size of 24 675 participants for further analyses (supplementary table A). In each individual study cohort, participants were followed until their first heart failure event within 15 years after the baseline examination, when follow-up time was censored. FHS participants were eligible for observation at the subsequent examination cycle if they had not developed heart failure. Incident heart failure during follow-up was independently adjudicated by experts. An explanation of how heart failure was identified and adjudicated in each cohort was previously reported,^16^ a summary of which is provided in the supplementary methods.

### Study definitions

At baseline, all participants had a detailed assessment of their medical history, drug treatment, physical examination, and fasting laboratory studies. All risk factors were evaluated and harmonized across cohorts.^16^ Body mass index was calculated as weight divided by height squared. We defined obesity as a body mass index of 30 or higher. Blood pressure was assessed as the average of two seated measurements. We defined diabetes mellitus as a fasting glucose of 126 mg/dL or higher, random glucose 200 mg/dL or higher, or the use of hypoglycemic drugs. Estimated glomerular filtration rate was calculated using the CKD-Epi formula.^17^ We defined atrial fibrillation as a past history or the presence of atrial fibrillation on electrocardiography.

During follow-up, the first occurrence of incident heart failure or death was recorded. All outcomes were adjudicated using established protocols by study investigators within each cohort after review of all available outpatient and hospital records as previously detailed.^16^ Heart failure was defined using a combination of signs and symptoms, and records were reviewed for assessment of left ventricular function at or around the time of the first presentation with heart failure. Each incident heart failure event was categorized as heart failure with preserved ejection fraction (left ventricular ejection fraction ≥50%), heart failure with reduced ejection fraction (left ventricular ejection fraction <50%), or unclassified (no left ventricular functional assessment available). Left ventricular function was ascertained by echocardiography in more than 85% of cases in all three cohorts. We considered participants to be unclassified if left ventricular function assessment at or around the time of heart failure presentation was unavailable.

### Statistical analyses

We summarized baseline characteristics by age group at study entry and presented them as means and standard deviations for continuous variables or numbers and percentages for categorical variables. The primary outcome was time until the first heart failure event. We depicted differences in survival between age groups at study entry and heart failure visually with Kaplan-Meier curves and examined them for group differences with the log-rank test. We used multivariable Cox models to examine the association of traditional risk factors with incident heart failure. Results of the Cox regression models show the mean hazard ratio with 95% confidence intervals as a measure of relative risk for each risk factor for developing heart failure, after control for other risk factors in the model, with a P value for statistical significance. Models were adjusted first for age and sex, and then additionally for ethnicity, body mass index, antihypertensive treatment, systolic blood pressure, smoking status, diabetes mellitus, previous myocardial infarction, and previous atrial fibrillation. For continuous risk factors, we presented effect sizes per one standard deviation difference. We did age-pooled analyses to examine multiplicative interaction terms between age as a linear variable and each risk factor. In addition, we used additive interaction analyses to study differences in the cumulative excess risk associated with risk factors with increasing age. We then used stratified Cox models by age group (young: <55 years, middle aged: 55-64 years, old: 65-74 years, elderly: ≥75 years) to examine the effect of risk factors on incident heart failure within an age group. To examine the absolute risk for developing heart failure associated with each risk factor, we show the incidence of heart failure per 1000 participant years, stratified by age groups and absence/presence of each risk factor. To further examine the association of these risk factors with heart failure, we dichotomized continuous variables (body mass index and systolic blood pressure) and then estimated the percentage of prevented cases if we were able to eliminate the risk factor from the population by using the previously proposed method of multivariable adjusted population attributable risk.^18^ All Cox models included a “strata” statement to allow baseline hazards to vary for each study cohort and account for possible differences between cohorts, different FHS examination cycles, and stratified recruitment in PREVEND. The proportionality assumption was met for Cox models. We considered a two sided P below 0.05 to be statistically significant. We used SAS 9.4 and R 3.6.0 for analyses.

### Patient and public involvement

No participants were involved in setting the research question or the outcome measures, nor were they involved in developing plans for design or implementation of the study. No participants were asked to advise on interpretation or writing up of results. We did not have access to patients or members of the public with the level of statistical or methodological expertise to analyze or interpret the present results.

## Results

### Baseline characteristics

Of the total 24 675 participants included for analysis, 9877 were included from FHS, 8115 from PREVEND, and 6683 from MESA. The mean age of the population was 56 (SD 14) years and 47% were men. In total, 11 599 (47%) of participants were classified as young (<55 years), 5587 (23%) as middle aged (55-64 years), 5190 (21%) as old (65-74 years), and 2299 (9%) as elderly (≥75 years) (table 1). The overall comorbidity burden was lower in younger participants and increased with age. Specifically, the prevalence of antihypertensive treatment, diabetes, previous myocardial infarction, and atrial fibrillation increased from 10%, 3%, 2%, and less than 1% respectively in young participants (<55 years) to 50%, 12%, 4%, and 6% in elderly participants (≥75 years).

**Table 1:** Clinical characteristics by age stratum. Values are numbers (percentages) unless stated otherwise

Parameter	Young (<55 years) (n=11 599)	Middle aged (55-64 years) (n=5587)	Old (65-74 years) (n=5190)	Elderly (≥75 years) (n=2299)	Total (n=24 675)
Mean (SD) age, years	44 (8)	60 (3)	69 (3)	80 (4)	56 (14)
Male sex	5542 (48)	2753 (49)	2527 (49)	964 (42)	11 696 (47)
Mean (SD) body mass index	26 (5)	28 (5)	28 (5)	27 (4)	27 (5)
Mean (SD) systolic blood pressure, mm Hg	121 (16)	131 (20)	138 (21)	141 (22)	129 (20)
Antihypertensive treatment	1109 (10)	1613 (29)	2175 (42)	1148 (50)	6045 (24)
Diabetes	348 (3)	516 (9)	636 (12)	264 (11)	1764 (7)
Current smoking	3777 (33)	1282 (23)	827 (16)	156 (7)	6042 (24)
Mean (SD) total cholesterol, mg/dL	205 (41)	216 (43)	213 (42)	203 (40)	209 (42)
Mean (SD) HDL cholesterol, mg/dL	51 (15)	51 (16)	50 (16)	51 (15)	51 (15)
Median (IQR) triglycerides, mg/dL	101 (72-179)	120 (86-174)	116 (84-163)	120 (84-168)	131 (77-160)
Prevalent myocardial infarction,	203 (2)	208 (4)	294 (6)	82 (4)	787 (3)
Prevalent atrial fibrillation	24 (<1)	91 (2)	137 (3)	127 (6)	339 (1)
Mean (SD) eGFR, mL/min/1.73 m^2^	82 (18)	74 (16)	69 (15)	59 (16)	76 (18)
Mean (SD) waist circumference, cm	90 (15)	97 (14)	98 (13)	98 (13)	94 (14)
Mean (SD) waist to hip ratio	0.88 (0.09)	0.93 (0.09)	0.94 (0.08)	0.95 (0.08)	0.91 (0.09)
White ethnicity	10 071 (87)	4385 (78)	3959 (76)	1730 (75)	20 145 (82)
Median (IQR) age at heart failure, years	58 (51-63)	72 (70-75)	81 (78-83)	89 (86-92)	68 (58-78)
Heart failure	138 (1)	293 (5)	538 (10)	412 (18)	1381 (6)
Heart failure with preserved ejection fraction	44 (<1)	97 (2)	184 (4)	179 (8)	504 (2)
Heart failure with reduced ejection fraction	91 (1)	184 (3)	319 (6)	168 (7)	762 (3)

eGFR=estimated glomerular filtration rate; HDL=high density lipoprotein; IQR=interquartile range.

### Cumulative incidence of heart failure according to age strata

Over a median follow-up of 12.7 (interquartile range 11.7-15.0) years, 1381 participants developed heart failure (table 1). We observed a total of 138 (1%) heart failure events among young participants compared with 412 (18%) heart failure events among elderly participants. The median age at onset of heart failure was 58 (51-63) years among young participants and 89 (86-92) years for elderly participants. Among participants who developed heart failure, 36% were classified as having heart failure with preserved ejection fraction and 55% as having heart failure with reduced ejection fraction; 9% were unclassified. Differences existed in heart failure subtypes across age categories. Among young participants who developed heart failure, 32% were classified as having heart failure with preserved ejection fraction and 66% as having heart failure with reduced ejection fraction (P<0.001; table 1). In elderly participants, 43% were classified as having heart failure with preserved ejection fraction and 41% as having heart failure with reduced ejection fraction. The proportion of unclassified heart failure was relatively higher in elderly participants (16%) than in young participants (2%).

### Predictors of incident heart failure by age

**Fig 1:**
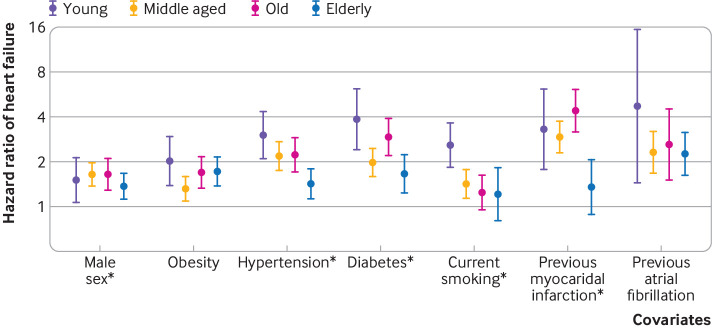
Forest plot depicting associations of risk factors with incident heart failure across age strata. *Age interaction term P<0.05

Age as a linear variable modified the association of some clinical risk factors and future development of heart failure. Specifically, we found significant interaction terms between age and sex, hypertension, diabetes, smoking, and previous myocardial infarction (P for interaction <0.05 for all) (fig 1; supplementary tables B and C). We observed significant additive interactions for sex, obesity, hypertension, previous myocardial infarction, and previous atrial fibrillation, such that with increasing age the additive excess risk for developing heart failure increased for these risk factors (supplementary table D). In analyses stratified by age group, hypertension was associated with a threefold higher risk of future heart failure in young participants (hazard ratio 3.02, 95% confidence interval 2.10 to 4.34) (table 2; supplementary table C), compared with a 1.4-fold increased risk among elderly participants (1.43, 1.13 to 1.81). Similarly, diabetes, smoking, and previous myocardial infarction conferred higher risk among younger participants, whereas effect sizes among elderly participants were much less pronounced for diabetes (hazard ratio: 3.86 (2.39 to 6.23) in young versus 1.66 (1.24 to 2.24) in elderly), smoking (2.58 (1.83 to 3.63) versus 1.21 (0.80 to 1.83)), and previous myocardial infarction (3.30 (1.77 to 6.14) versus 1.35 (0.89 to 2.08)). By contrast, we did not find that age modified the association of body mass index or obesity with future heart failure. The overall predictive accuracy for the models declined with increasing age, with a C index of 0.79 in young participants, 0.74 in middle aged participants, 0.62 in old participants, and 0.63 in elderly participants. In secondary analyses, we added estimated glomerular filtration rate to the model; it was not associated with incident heart failure (hazard ratio 0.97, 0.89 to 1.06) and did not show a significant interaction with age (P for interaction >0.1). The absolute risk for heart failure was lower in younger participants and increased with older age (supplementary table E). For example, younger people with diabetes had more a more than sixfold greater incidence of heart failure than did those without diabetes (6.0 *v* 0.8 events per 1000 participant years). By contrast, the overall incidence of heart failure was greater but the difference between those with and without diabetes was less pronounced among elderly participants (26.7 *v* 19.7 events per 1000 participant years).

**Table 2:** Age stratified associations of risk factors with incident heart failure

Parameter	Young (<55 years); 138 events/11 599 people				Middle aged (55-64 years); 293 events/5587 people				Old (65-74 years); 538 events/5190 people				Elderly (≥75 years); 412 events/2299 people		
	HR (95% CI)	P value	PAR, %		HR (95% CI)	P value	PAR, %		HR (95% CI)	P value	PAR, %		HR (95% CI)	P value	PAR, %
Male sex	1.51 (1.07 to 2.15)	0.02	21		1.65 (1.29 to 2.10)	<0.001	25		1.65 (1.38 to 1.97)	<0.001	24		1.37 (1.11 to 1.68)	0.003	13
Obesity	2.03 (1.39 to 2.96)	<0.001	21		1.69 (1.32 to 2.16)	<0.001	17		1.32 (1.09 to 1.60)	0.004	8		1.73 (1.38 to 2.15)	<0.001	13
Hypertension	3.02 (2.10 to 4.34)	<0.001	35		2.23 (1.72 to 2.91)	<0.001	40		2.19 (1.76 to 2.73)	<0.001	44		1.43 (1.13 to 1.81)	0.003	23
Diabetes	3.86 (2.39 to 6.23)	<0.001	14		2.93 (2.20 to 3.90)	<0.001	16		1.98 (1.59 to 2.46)	<0.001	12		1.66 (1.24 to 2.24)	<0.001	7
Current smoking	2.58 (1.83 to 3.63)	<0.001	32		1.25 (0.96 to 1.63)	0.1	5		1.43 (1.15 to 1.77)	0.001	6		1.21 (0.80 to 1.83)	0.36	1
Previous MI	3.30 (1.77 to 6.14)	<0.001	6		4.40 (3.17 to 6.10)	<0.001	13		2.92 (2.28 to 3.74)	<0.001	11		1.35 (0.89 to 2.08)	0.15	1
Previous AF	4.74 (1.44 to 15.6)	0.01	2		2.62 (1.51 to 4.52)	<0.001	3		2.31 (1.67 to 3.21)	<0.001	5		2.26 (1.62 to 3.15)	<0.001	5
Cumulative PAR, %	75				77				76				53		

AF=atrial fibrillation; HR=hazard ratio; MI=myocardial infarction; PAR=population attributable risk.

### Population attributable risk for future heart failure across age groups

**Fig 2:**
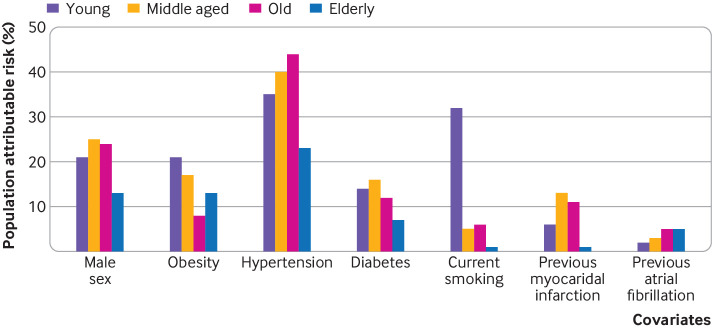
Bar plot showing population attributable risk for risk factors and incident heart failure across age categories. AF=atrial fibrillation; MI=myocardial infarction

When we investigated the population attributable risk for each risk factor, a greater proportion of the risk for developing heart failure was explained by traditional risk factors in younger patients (fig 2; table 2). For example, the population attributable risk for obesity was 21% among young participants, 17% among middle aged participants, 8% among old participants, and 13% among elderly participants. Similarly, the population attributable risk for smoking was 32% among young participants, 5% among middle aged participants, 6% among old participants, and 1% elderly participants. The population attributable risk for diabetes mellitus ranged from 14% in young participants to 7% in elderly participants. By contrast, the highest population attributable risk for future heart failure was for hypertension across all age groups (35% in young, 40% in middle aged, 44% in old, and 23% in elderly participants). When examining the total population attributable risk for future heart failure conferred by traditional risk factors combined (sex, hypertension, obesity, diabetes, smoking, previous myocardial infarction, and previous atrial fibrillation), we found that overall population attributable risk was highest and explained 75% of heart failure risk in young participants, compared with only 53% population attributable risk in elderly participants.

### Effect of age on risk factors for heart failure with preserved ejection fraction and heart failure with reduced ejection fraction

When examining heart failure subtypes, we found that the association between many traditional risk factors and incident heart failure with reduced ejection fraction as well as heart failure with preserved ejection fraction were also modified by age (supplementary table B). For incident heart failure with reduced ejection fraction, we found significant interaction terms between age and hypertension, diabetes, myocardial infarction, and atrial fibrillation (P for interaction for all <0.05). In stratified analyses (supplementary table C), these risk factors conferred a greater risk for incident heart failure with reduced ejection fraction in young compared with elderly participants (hazard ratio 2.76 (1.77 to 4.32) in young versus 1.54 (1.06 to 2.23) in elderly participants for hypertension; 3.28 (1.75 to 6.13) versus 1.62 (1.02 to 2.56) for diabetes; 3.59 (1.74 to 7.41) versus 2.03 (1.17 to 3.54) for myocardial infarction; 4.91 (1.14 to 21.06) versus 1.52 (0.87 to 2.65) for atrial fibrillation). For incident heart failure with preserved ejection fraction, we found significant interactions between age and hypertension, diabetes, and myocardial infarction (P for interaction for all <0.05; supplementary table B). Compared with elderly participants, these risk factors conferred a greater risk for incident heart failure with preserved ejection fraction (supplementary table C; supplementary figure A) in young participants (hazard ratio 3.06 (1.60 to 5.86) in young versus 1.14 (0.81 to 1.60) in elderly participants for hypertension; 4.94 (2.24 to 10.88) in young versus 1.32 (0.81 to 2.18) in elderly participants for diabetes; 2.85 (0.84 to 9.69) in young versus 1.51 (0.88 to 2.60) in old participants for myocardial infarction). Similarly, population attributable risk for obesity, hypertension, diabetes, and smoking history was greatest among young participants and declined with increasing age for both incident heart failure with reduced ejection fraction and heart failure with preserved ejection fraction (supplementary table C; supplementary figure B).

## Discussion

In this study of 24 675 participants, we showed important age differences in the effect of traditional risk factors on the development of future heart failure. Specifically, our findings support the idea that modifiable clinical risk factors for heart failure carry both a stronger relative risk and greater population attributable risk among young people than older people. In total, 75% of total attributable risk for heart failure was captured by traditional risk factors among young participants, compared with 53% of risk among elderly participants. Similarly, the contribution of combined risk factors to future risk of heart failure as assessed by the C statistic was greater among younger than older participants. These age differences in the effect of risk factors on heart failure seemed to extend to both heart failure with preserved ejection fraction and heart failure with reduced ejection fraction. Interestingly, we found that more than 30% of participants who developed heart failure below the baseline age of 65 were classified as having heart failure with preserved ejection fraction, often considered a disease of older people. The absolute risk for each individual risk factor increased with age. These findings highlight the importance of not only considering risk factor modification in older people but also extending preventive efforts to younger people at high risk for developing heart failure.

### Comparison with other studies

Despite evidence of a relative stabilization of the incidence of heart failure, its prevalence continues to increase owing to an enlarging population of people at risk. This includes young people, among whom a rapid increase in the prevalence of risk factors for developing heart failure, such as obesity and diabetes, forebodes a potential epidemic. The increased burden of metabolic syndrome in younger people has been linked to a relative increase of incident heart failure among young compared with older people in two separate studies.^5^ ^6^ Data from patients with prevalent heart failure show that a significant proportion of patients with heart failure are young (<65 years), even among those with heart failure with preserved ejection fraction.^7^ ^8^ ^9^ We observed a higher incidence of new onset of heart failure with preserved ejection fraction in elderly participants, whereas new onset of heart failure with reduced ejection fraction was more common in younger participants. In a combined cohort study of FHS, PREVEND, and CHS, older age was more strongly associated with new onset of heart failure with preserved ejection fraction than of heart failure with reduced ejection fraction. However, in the same study, the accuracy of two separately developed prediction models did not differ for predicting new onset of heart failure with preserved ejection fraction or heart failure with reduced ejection fraction.^16^ This might reflect differences in etiology, whereby hypertensive heart disease is more common in heart failure with preserved ejection fraction and ischemic heart disease is more common in heart failure with reduced ejection fraction.^19^ Our data are consistent with previous studies and extend them by comparing the age dependent association of several common risk factors with incident heart failure in a pooled analysis of three large community based cohorts. Furthermore, we observed similar age dependent associations for incident heart failure with reduced ejection fraction and heart failure with preserved ejection fraction.

We found differential associations of common risk factors for heart failure by age, whereby people who were 55 years or younger with hypertension, diabetes, smoking, and past myocardial infarction had a significantly greater relative risk for heart failure within their age group, compared with older people with risk factors. This is consistent with previous evidence of a smaller excess risk for cardiovascular disease associated with hypertension and diabetes with older age.^20^ ^21^ ^22^ ^23^ ^24^ ^25^ ^26^ In the CArdiovascular research using LInked Bespoke studies and Electronic health Records (CALIBER) study, the relative risk associated with hypertension for 12 cardiovascular diseases decreased with increasing age.^20^ Similarly, data from the National Diabetes Services Scheme (NDSS) and Swedish National Diabetes Register suggest that younger onset of type 2 diabetes increases mortality risk.^23^ ^26^ Previous studies were limited by focusing primarily on macrovascular disease, by not having a validated diagnosis of heart failure, and by focusing on one risk factor or a limited number of risk factors.^21^ ^23^ ^25^ ^27^ We extend these findings in several important ways by investigating age dependent associations specifically for heart failure in a large richly phenotyped cohort, including a well validated outcome, and showing that age dependent differences in the relative risk for developing heart failure exist for multiple common risk factors.

The much greater relative risk associated with risk factors in younger participants and the potential benefit of treatment and lifestyle changes should be interpreted in light of the lower absolute risk and greater population attributable risk of risk factors in young people. The lower absolute risk for each risk factor suggests that the number needed to treat for public health interventions is larger in younger than older people. Young people with mild hypertension without target organ damage have a lower 10 year risk of cardiovascular disease than older people.^28^ The higher population attributable risk highlights that a larger proportion of the risk for heart failure in young people will be sensitive to intervention, compared with older people. Importantly, the potential number of disease-free life years lost is more substantial in young people.^27^ ^29^ Thus, longer time horizons might be necessary to show benefits of targeted preventive efforts in young people, and this should take into account the potential number of disease-free life years saved.

A discussion on possible reasons for age dependent association of risk factors with incident heart failure is warranted. A low baseline risk for heart failure among community based young adults, particularly those without known risk factors, may explain the higher relative risk associated with a particular risk factor among the young; that is, the risk conferred by a risk factor in a young person relative to a healthy young adult (without that risk factor) may be greater than that in an older person whose baseline risk for heart failure is higher. Furthermore, the lower population attributable risk and predictive accuracy of common risk factors in older participants highlights the greater complexity of heart failure risk in older people and might also suggest that other etiologies, risk factors, and lifestyle factors not captured in this study might be associated with heart failure in older people. The population attributable risk for obesity was higher in young than in old people. This is consistent with data on prevalent heart failure, which show that patients with obesity are more likely to be younger.^7^ ^9^ Some evidence suggests that obesity in younger patients with prevalent heart failure is associated with worse mortality, highlighting the possible importance of obesity in developing the disease at an early age.^9^ Although we accounted for known risk factors in multivariable modeling, unknown or unmeasured age related processes are likely to play a role, such as increased oxidative stress, DNA damage events and impaired repair, and slow accumulation of myocardial fibrosis over the lifespan.^30^ This might also explain the age dependent increase in the proportion of participants with heart failure with preserved ejection fraction, which is often considered a disease of older people and associated with increased myocardial fibrosis.^16^ ^31^ Although duration of disease may contribute, several studies in diabetes have shown that the age dependent association with worse outcomes persisted after control for disease duration.^25^ ^32^ Another plausible explanation for our findings might be differences in risk factor control or severity. People with early onset of diabetes more frequently have concomitant obesity and poor risk factor control.^33^ Similarly, control of hypertension was worse in younger than older men in a study among 152 561 patients with incident hypertension.^34^ However, risk factor control can also be problematic in older people, in whom resistant hypertension is more prevalent.^35^ Lastly, patients with early onset of risk factors such as diabetes, obesity, and hypertension more often have a lower socioeconomic status, which is independently associated with worse outcomes, poorer healthcare seeking behavior, and worse risk factor control. The higher combined population attributable risk of multiple common risk factors for heart failure, especially in young people, emphasizes the need for a holistic approach to the prevention of heart failure. Regardless of the explanation, our findings advocate for efforts to prevent heart failure to extend to younger people to reduce the lifetime risk for developing heart failure. Aggressive risk factor control in younger, as in older, adults may include strict blood pressure control, prescription of statins, good glycemic control, and supporting sustainable weight loss strategies.

### Strengths and weaknesses of study

Strengths of this study include the large number of participants, the rigorous phenotyping of the cohorts, and a well validated endpoint. The study also has several limitations. We did not have information available on duration or control of risk factors or on adherence to treatment; these and other unmeasured factors may cause residual confounding. By definition, participants classified as having heart failure with reduced ejection fraction and heart failure with preserved ejection fraction were only those who underwent left ventricular function assessment at or around the time of presentation with heart failure. Although we included some of the largest population based samples available to date, our secondary analyses were still relatively underpowered owing to lower event rates in younger age groups. We corrected for differences in stratified recruitment in PREVEND and possible differences in design between cohorts by including specific strata in our Cox models, but we cannot exclude the possibility that our results were affected by these differences. Although we included ethnicity in our multivariable models, we did not have adequate power to do ethnicity specific analyses. Extrapolation of our results to more geographically and ethnically diverse populations should be done with caution. Socioeconomic status was not available in the pooled dataset, and whether the observed associations can be extrapolated to populations of lower socioeconomic status is unclear. We investigated the association of current smoking with incident heart failure, but we were not able to account for number of pack years. Data on cardiovascular drugs such as lipid lowering therapy were not available in the pooled dataset, so we were unable to account for this in the multivariable prediction model. This might have influenced the decrease in predictive accuracy of the models with older age. Other key lifestyle risk factors such as cardiorespiratory fitness and exercise are associated with new onset of heart failure,^36^ ^37^ ^38^ but they were not captured in our study. Age dependent differences for these risk factors merit further study.

### Conclusion and policy implications

In sum, despite a lower incidence of heart failure among younger compared with older people, we have shown that modifiable clinical risk factors for heart failure carry both a stronger relative risk and a greater population attributable risk among young people than older people. These findings highlight the importance of preventive efforts across the adult life course.

## What is already known on this topic

The incidence of heart failure is lower in younger than in older people; few studies have investigated age differences in risk factors associated with incident heart failure
A study using linked electronic health records found a decrease in the relative risk of increased blood pressure with the incidence of 12 cardiovascular diseases including heart failure
Studies in patients with prevalent heart failure highlighted that younger patients (≤55 years) were more likely to be obese, to be of male sex, or to have a history of diabetes

## What this study adds

Hypertension, diabetes, smoking history, and previous myocardial infarction conferred a greater relative risk of heart failure in younger than in older people
Risk factors had greater discriminatory value in predicting new onset of heart failure in younger than in older people
A greater proportion of the overall population attributable risk was explained by diabetes, smoking history, and previous myocardial infarction in younger than in older people

## Web extra

Extra material supplied by authors

Web appendix: Supplementary materials

## Notes

Contributors: JT, SMAP, SJS, RAB, CSPL, and JEH contributed equally to this work. JT, SMAP, CSPL, and JEH designed the study. All authors were involved in analyzing and interpreting the data. JT and SMAP drafted the manuscript. ESL, NBA, MJB, RTG, HLH, DEL, DL, VSR, PH, WHG, MGL, SJS, RAB, CSPL, and JEH critically revised the manuscript for important intellectual content. JT and SMAP did the statistical analysis. JEH obtained funding and provided administrative support. CSPL and JEH supervised the study. The corresponding author attests that all listed authors meet authorship criteria and that no others meeting the criteria have been omitted. JT, SMAP, and JEH are the guarantors.

Ethical approval: Written informed consent was obtained, and institutional review board approval was obtained at all participating institutions.

Data sharing: Participant level data are available at the database of Genotypes and Phenotypes (https://www.ncbi.nlm.nih.gov/gap/) and BioLINCC (https://biolincc.nhlbi.nih.gov/home/) and are available on request for replication of results by contacting the corresponding author.

The lead authors affirm that the manuscript is an honest, accurate, and transparent account of the study being reported; that no important aspects of the study have been omitted; and that any discrepancies from the study as planned (and, if relevant, registered) have been explained.

Dissemination to participants and related patient and public communities: Results of this study will be disseminated to study participants and the general public via emailed newsletters relevant to the individual cohorts, institutional and cohort specific websites, and social media channels where available.

Provenance and peer review: Not commissioned; externally peer reviewed.
