# Supplementary material for: Age dependent associations of risk factors with heart failure: pooled population based cohort study
Source: BMJ. 2021 Mar 23;372:n461. doi: 10.1136/bmj.n461 (PMC7986583; doi:10.1136/bmj.n461)
Supplement: Supplementary file 1 — Web appendix: Supplementary materials [file troj061141.ww1.pdf]

Supplementary material.

Supplementary Table A: calculation of study sample

Exclusion information		
**Exclusion Criteria**	**Frequency**	**%**
Inclusion Sample	*24675*	*95.05*
Prevalent HF	195	0.75
No follow-up	27	0.1
missing covariates*	1063	4.09
Starting Sample	*25960*	*100*

Abbreviations: HF, heart failure

Supplementary Table B: Age-interactions of risk factors with incident heart failure, heart failure with reduced ejection fraction and heart failure with preserved ejection fraction in pooled models.

Heart Failure

n=1381/24675

Parameter	HR (95%CI)	P-value	Age*covariate interaction term HR (95% CI) *	Age* interaction p-value
Age	3.23 (2.93-3.56)	<0.001		
Men (vs. women)	1.54 (1.38-1.72)	<0.001	0.84 (0.73-0.97)	0.02
Obesity	1.65 (1.47-1.85)	<0.001	0.93 (0.80-1.10)	0.38
Hypertension	1.98 (1.74-2.26)	<0.001	0.75 (0.64-0.88)	<0.001
Diabetes	2.16 (1.87-2.48)	<0.001	0.63 (0.51-0.78)	<0.001
Current Smoking	1.54 (1.34-1.76)	<0.001	0.79(0.68-0.95)	0.01
Past MI	2.67 (2.26-3.17)	<0.001	0.62 (0.49-0.77)	<0.001
Past AF	2.22 (1.79-2.74)	<0.001	0.74 (0.53-1.03)	0.07

Heart Failure w/ **Preserved Ejection Fraction**

n = 504 / 24675

Parameter	HR (95%CI)	P-value	Age*covariate interaction term HR (95% CI) *	Age* interaction p-value
Age	3.73 (3.16-4.39)	<0.001		
Men (vs. women)	0.89 (0.74-1.08)	0.23	0.98 (0.77-1.24)	0.85
Obesity	1.76 (1.45-2.13)	<0.001	0.98 (0.76-1.27)	0.89
Hypertension	1.80 (1.46-2.23)	<0.001	0.65(0.50-0.84)	<0.001
Diabetes	2.01 (1.58-2.55)	<0.001	0.56 (0.39-0.80)	<0.001
Current Smoking	1.31 (1.03-1.68)	0.03	0.79(0.57-1.08)	0.14
Past MI	1.60 (1.13-2.28)	0.01	0.54 (0.34-0.86)	0.01
Past AF	2.84 (2.00-4.02)	<0.001	1.23 (0.68-2.23)	0.49

Heart Failure w/ Reduced Ejection Fraction

n = 762 / 24675

Parameter	HR (95%CI)	P-value	Age*covariate interaction term HR (95% CI) *	Age* interaction p-value
Age	2.74 (2.42-3.11)	<0.001		
Men (vs. women)	2.27 (1.94-2.65)	<0.001	0.84 (0.69-1.02)	0.07
Obesity	1.64 (1.40-1.92)	<0.001	0.93(0.76-1.15)	0.52
Hypertension	2.08 (1.75-2.48)	<0.001	0.80(0.66-0.98)	0.03
Diabetes	2.19 (1.81-2.65)	<0.001	0.65 (0.49-0.85)	<0.001
Current Smoking	1.73 1.46-2.06)	<0.001	0.89(0.72-1.11)	0.29
Past MI	3.44 (2.80-4.22)	<0.001	0.70 (0.54-0.92)	0.01
Past AF	1.87 (1.39-2.50)	<0.001	0.48 (0.32-0.74)	<0.001

*Age was included on a linear scale per 1-standard deviation difference.

Abbreviations: AF, atrial fibrillation; HR, hazard ratio; MI, myocardial infarction; PAR, population attributable risk

Supplementary Table C: age stratified associations of risk factors with incident heart failure with reduced ejection fraction and heart failure with preserved ejection fraction.

HFpEF

	Young (age: <55)			Middle-aged (age: 55-64) **			Old (age: 65-74)			Elderly (age ≥75)		
n events/ n	44/11599			97/5587			184/5190			179/2299		
**Parameter**	**HR**	**P-value**	**PAR**	**HR**	**P-value**	**PAR**	**HR**	**P-value**	**PAR**	**HR**	**P-value**	**PAR**
Female Sex	1.37 (0.75-2.51)	0.31	15%	1.14 (0.76-1.70)	0.54	6%	1.08 (0.80-1.46)	0.61	4%	1.08 (0.67-1.29)	0.66	5%
Obesity	2.23 (1.14-4.36)	0.02	25%	1.53 (1.01-2.37)	0.05	15%	1.44 (1.05-1.98)	0.02	11%	1.92 (1.39-2.67)	<0.001	15%
Hypertension	3.06 (1.60-5.86)	<0.001	36%	2.15 (1.37-3.39)	0.001	38%	2.38 (1.62-3.47)	<0.001	47%	1.14 (0.81-1.60)	0.46	9%
Diabetes	4.94 (2.24-10.88)	<0.001	18%	3.12 (1.91-5.08)	<0.001	18%	1.78 (1.21-2.60)	0.003	9%	1.32 (0.81-2.18)	0.27	4%
Current Smoking	2.11 (1.15-3.88)	0.02	25%	1.02 (0.63-1.65)	0.93	1%	1.32 (0.88-1.96)	0.18	4%	1.11 (0.57-2.19)	0.76	1%
Past MI	2.85 (0.84-9.69)	0.09	4%	3.27 (1.73-6.19)	<0.001	7%	1.51 (0.88-2.60)	0.14	3%	[redacted]		
Past AF	5.18 (0.66-40.54)	0.12	2%	1.42 (0.34-5.98)	0.61	1%	2.66 (1.48-4.81)	0.001	5%	3.42 (2.13-5.48)	<0.001	8%
Cumulative PAR			75%			60%			81%			43%

* not shown but additionally adjusted for caucasian race

* due to low event rates within the elderly for HFpEF and past mi, past mi was not considered for the MV model.

HFrEF

	Young (age: <55)			Middle-aged (age: 55-64)			Old (age: 65-74) *			Elderly (age ≥75)		
n events/ n	91/11599			184/5587			319/5190			168/2299		
**Parameter**	**HR**	**P-value**	**PAR**	**HR**	**P-value**	**PAR**	**HR**	**P-value**	**PAR**	**HR**	**P-value**	**PAR**
Male Sex	2.23 (1.41-3.51)	<0.001	39%	2.44 (1.75-3.40)	<0.001	43%	2.46 (1.92-3.16)	<0.001	42%	1.85 (1.34-2.55)	<0.001	25%
Obesity	2.02 (1.26-3.23)	0.003	20%	1.71 (1.25-2.33)	<0.001	17%	1.30 (1.01-1.68)	0.04	7%	1.78 (1.26-2.52)	0.001	13%
Hypertension	2.76 (1.77-4.32)	<0.001	32%	2.23 (1.60-3.11)	<0.001	40%	2.15 (1.62-2.86)	<0.001	43%	1.54 (1.06-2.23)	0.02	27%
Diabetes	3.28 (1.75-6.13)	<0.001	11%	2.89 (2.01-4.16)	<0.001	15%	2.07 (1.56-2.74)	<0.001	13%	1.62 (1.02-2.56)	0.04	7%
Current Smoking	2.77 (1.81-4.22)	<0.001	34%	1.45 (1.05-2.01)	0.02	10%	1.50 (1.14-1.97)	0.004	7%	1.50 (0.84-2.69)	0.17	3%
Past MI	3.59 (1.74-7.41)	<0.001	8%	4.72 (3.19-7.00)	<0.001	15%	3.65 (2.72-4.89)	<0.001	15%	2.03 (1.17-3.54)	0.01	4%
Past AF	4.91 (1.14-21.06)	0.03	2%	2.87 (1.54-5.33)	<0.001	4%	1.97 (1.29-3.02)	0.002	4%	1.52 0.87-2.65)	0.14	3%
Cumulative PAR			80%			87%			78%			64%

* not shown but additionally adjusted for caucasian race

Abbreviations: AF, atrial fibrillation; HFpEF, heart failure with preserved ejection fraction; HFrEF, heart failure with reduced ejection fraction; HR, hazard ratio; MI, myocardial infarction; PAR, population attributable risk

Supplementary Table D: Results of additive interaction analyses.

Age groups	**RERI**	**95% CI**	**P-value**	**Factor**
<55 years	ref	ref	ref	Male
55-64	1.74	0.61 to 2.87	0.003	
65-74	3.77	1.97 to 5.58	<0.001	
≥75 years	2.76	-1.39 to 6.70	0.192	
<55 years	ref	ref	ref	Obese
55-64	1.5	-0.17 to 3.16	0.078	
65-74	1.14	-1.21 to 3.50	0.340	
≥75 years	16.11	6.66 to 25.55	0.001	
<55 years	ref	ref	ref	HTN
55-64	3.77	1.71 to 5.82	<0.001	
65-74	8.31	4.76 to 11.86	<0.001	
≥75 years	15.21	4.44 to 25.98	0.006	
<55 years	ref	ref	ref	DM
55-64	3.39	-0.25 to 7.04	0.068	
65-74	2.78	-1.06 to 6.62	0.156	
≥75 years	4.74	-5.54 to 15.01	0.366	
<55 years	ref	ref	ref	Smoke
55-64	0.17	-1.45 to 1.78	0.840	
65-74	3.23	0.26 to 6.20	0.028	
≥75 years	4.46	-9.31 to 18.23	0.517	
<55 years	ref	Ref	ref	Previous MI
55-64	8.29	3.34 to 13.24	0.001	
65-74	10.88	5.28 to 16.48	<0.001	
≥75 years old	6.33	-7.05 to 19.70	0.354	
<55 years	ref	Ref	ref	Previous AF
55-64	4.53	-3.05 to 12.56	0.269	

65-74	5.98	-1.59 to 13.55	0.122	
≥75 years	24.06	7.20 to 40.92	0.005	

Abbreviations: CI, confidence interval; RERI, Relative excess risk due to interaction

Supplementary Table E: Incidence of heart failure, HFrEF and HFpEF in individuals with listed risk factors stratified to age.

	**Heart failure**							
	**Young (age: <55)**		**Middle-aged (age: 55-64)**		**Old (age: 65-74)**		**Elderly (age ≥75)**	
**Parameter**	No	Yes	No	Yes	No	Yes	No	Yes
	Events/1000 PY (95%CI)							
Men	0.66 (0.48 to 0.84)	1.24 (0.98 to 1.51)	2.95 (2.39 to 3.51)	5.65 (4.84 to 6.46)	7.01 (6.07 to 7.94)	12.33 (10.98 to 13.68)	18.96 (16.54 to 21.38)	22.21 (18.93 to 25.50)
Obesity	0.68 (0.53 to 0.82)	2.11 (1.56 to 2.66)	3.49 (2.97 to 4.00)	6.35 (5.20 to 7.51)	8.73 (7.84 to 9.62)	11.53 (9.78 to 13.29)	18.13 (16.07 to 20.20)	28.82 (23.55 to 34.08)
Hypertension	0.55 (0.42 to 0.68)	2.48 (1.91 to 3.05)	2.20 (1 to72 to 2.67)	6.60 (5.71 to 7.49)	4.90 (3.96 to 5.84)	12.13 (10.99 to 13.27)	15.06 (12.02 to 18.11)	22.51 (20.03 to 24.99)
Diabetes	0.79 (0.64 to 0.93)	6.02 (3.65 to 8.37)	3.58 (3.11 to 4.05)	11.64 (8.83 to 14.45)	8.33 (7.54 to 9.13)	18.28 (14.95 to 21.61)	19.50 (17.48 to 21.52)	26.74 (19.67 to 33.81)
Smoker	0.65 (0.49 to 0.81)	1.54 (1.19 to 1.89)	3.91 (3.38 to 4.43)	5.43 (4.27 to 6.61)	8.76 (7.93 to 9.59)	13.55 (11.00 to 16.09	20.12 (18.12 to 22.12)	22.09 (13.43 to 30.75)
Past MI	0.87 (0.72 to 1.02)	5.07 (2.20 to 7.94)	3.62 (3.17 to 4.08)	23.41 (16.98 to 29.83)	8.19 (7.43 to 8.95)	37.95 (30.15 to 45.75)	19.43 (17.50 to 21.38)	48.27 (30.06 to 66.47)
Past AF	0.92 (0.76 to 1.07)	9.87 (0.0 to 21.0)	4.06 (3.59 to 4.54)	26.13 (12.9 to 39.35)	8.87 (8.09 to 9.65)	35.86 (25.14 to 46.57)	18.71 (16.79 to 20.62)	60.06 (42.51 to 77.61)
	**Heart failure with reduced ejection fraction**							
Men	0.34 (0.21 to 0.47)	0.93 (0.69 to 1.15)	1.39 (1.01 to 1.78)	4.05 (3.36 to 4.74)	3.16 (2.54 to 3.79)	8.49 (7.37 to 9.61)	6.59 (5.16 to 8.02)	10.86 (8.56 to 13.15)
Obesity	0.46 (0.34 to 0.58)	1.32 (0.88 to 1.76)	2.25 (1.83 to 2.66)	3.83 (2.94 to 4.73)	5.33 (4.63 to 6.02)	6.39 (5.09 to 7.70)	7.39 (6.07 to 8.71)	11.78 (8.41 to 15.14)
Hypertension	0.38 (0.27 to 0.49)	1.56 (1.11 to 2.01)	1.38 (1.00 to 1.76)	4.14 (3.44 to 4.84)	2.92 (2.19 to 3.65)	7.18 (6.30 to 8.06)	5.93 (4.02 to 7.84)	9.27 (7.69 to 10.86)
Diabetes	0.53 (0.42 to 0.66)	3.37 (1.61 to 5.13)	2.27 (1.90 to 2.65)	7.06 (4.87 to 9.24)	4.90 (4.29 to 5.51)	11.19 (8.59 to 13.79)	7.92 (6.63 to 9.21)	11.18 (6.61 to 15.75)

Smoker	0.41 (0.28 to 0.53)	1.06 (0.76 to 1.35)	2.33 (1.92 to 2.73)	3.87 (2.88 to 4.85)	5.05 (4.42 to 5.68)	8.95 (6.88 to 11.01)	8.06 (6.79 to 9.33)	11.49 (5.24 to 17.73)
Past MI	0.56 (0.44 to 0.69)	3.81 (1.32 to 6.29)	2.20 (1.84 to 2.56)	16.98 (11.50 to 22.45)	4.56 (3.99 to 5.13)	29.19 (22.35 to 36.02)	7.62 (6.41 to 8.84)	30.39 (15.94 to 44.84)
Past AF	0.60 (0.48 to 0.73)	6.58 (0 to 15.69)	2.51 (2.14 to 2.89)	20.90 (9.08 to 32.73)	5.27 (4.67 to 5.87)	20.85 (12.68 to 20.02)	7.75 (6.52 to 8.98)	21.35 (10.89 to 31.82)
	**Heart failure with preserved ejection fraction**							
Men	2.12 (1.79 to 2.44)	3.02 (2.61 to 2.43)	6.96 (6.10 to 7.82)	12.67 (11.45 to 13.88)	17.21 (15.75 to 18.6&)	29.20 (27.12 to 31.27)	49.34 (45.43 to 53.24)	63.63 (58.07 to 69.18)
Obesity	2.29 (2.02 to 2.56)	3.69 (2.96 to 4.42)	9.77 (8.91 to 10.64)	9.47 (8.06 to 10.89)	23.28 (21.83 to 24.73)	20.91 (18.55 to 23.27)	57.52 (53.85 to 61.19)	44.10 (37.59 to 50.62)
Hypertension	2.15 (1.88 to 2.41)	4.11 (3.38 to 4.84)	7.78 (6.88 to 8.68)	11.89 (10.70 to 13.09)	19.28 (17.41 to 21.15)	24.70 (23.08 to 26.33)	52.72 (47.03 to 58.42)	55.85 (51.96 to 59.75)
Diabetes	2.38 (1.13 to 2.64)	7.95 (5.23 to 10.66)	9.11 (8.37 to 9.85)	16.23 (12.91 to 19.55)	22.05 (20.76 to 23.35)	27.73 (23.63 to 31.83)	54.45 (51.07 to 57.83)	58.83 (48.35 to 69.31)
Smoker	1.77 (1.51 to 2.03)	4.18 (3.60 to 4.76)	7.76 (7.01 to 8.50)	16.52 (14.48 to 18.56)	19.67 (18.42 to 20.91)	41.01 (36.58 to 45.43)	52.51 (49.27 to 55.75)	95.43 (77.43 to 113.42)
Past MI	2.50 (2.24 to 2.76)	5.07 (2.20 to 7.95)	9.26 (8.53 to 9.99)	22.95 (16.59 to 29.31)	21.59 (20.36 to 22.83)	47.54 (38.81 to 56.27)	53.97 (50.73 to 57.21)	87.59 (63.07 to 112.12)
Past AF	2.54 (2.28 to 2.80)	3.29 (0 to 9.74)	9.67 (8.84 to 10.41)	12.19 (3.16 to 21.23)	22.13 (20.89 to 23.37)	48.37 (35.92 to 60.81)	52.96 (49.74 to 56.18)	105.44 (82.19 to 128.69)

Abbreviations: AF, atrial fibrillation; MI, myocardial infarction.

Supplementary Figure A: Forest plots depicting associations of risk factors for incident HFpEF (A) and HFrEF (B) across age groups. * signifies an age interaction term P <0.05.

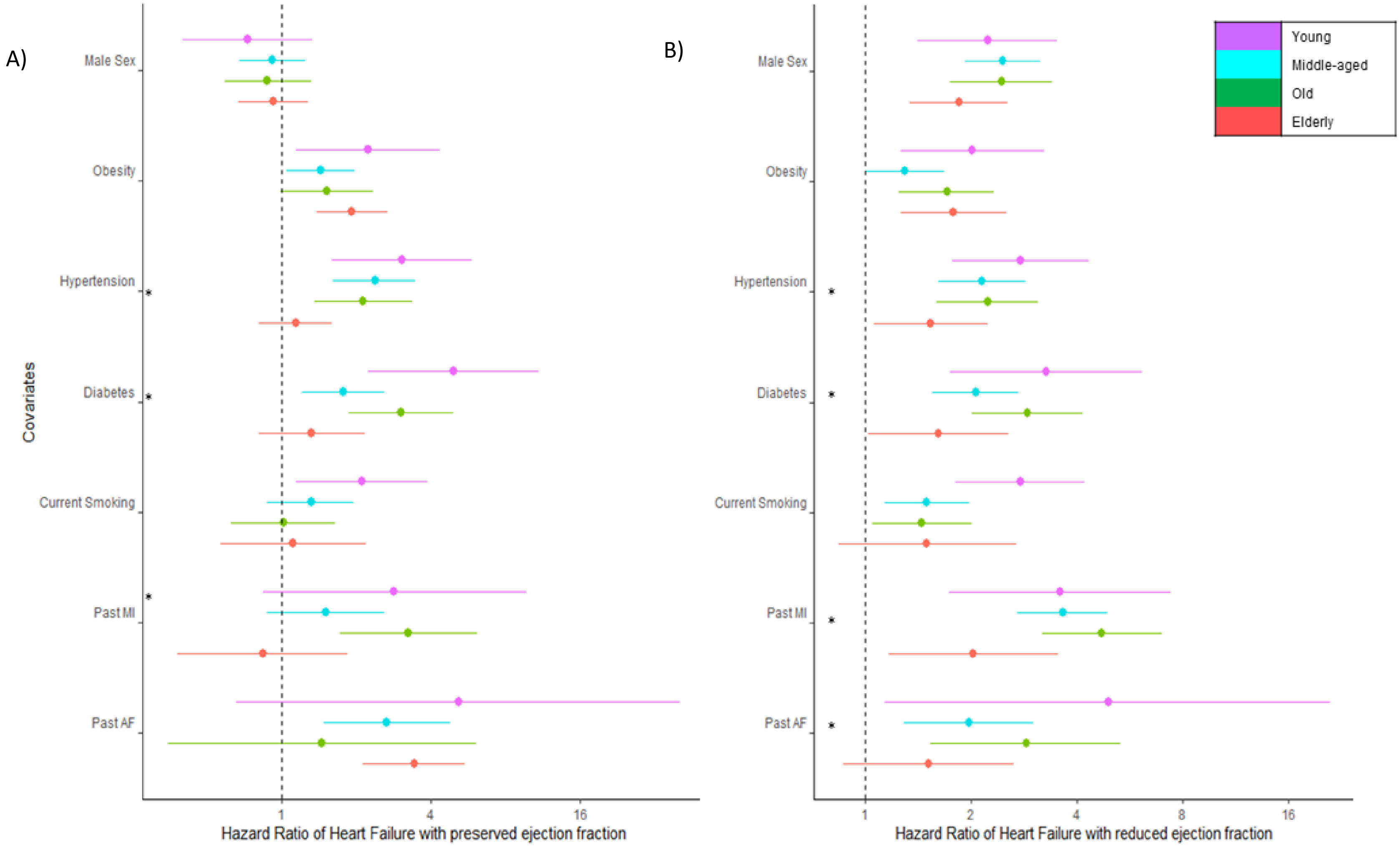

Supplementary Figure B: Bar plot showing the population attributable risk for risk factors and incident HFrEF and HFpEF across age categories.

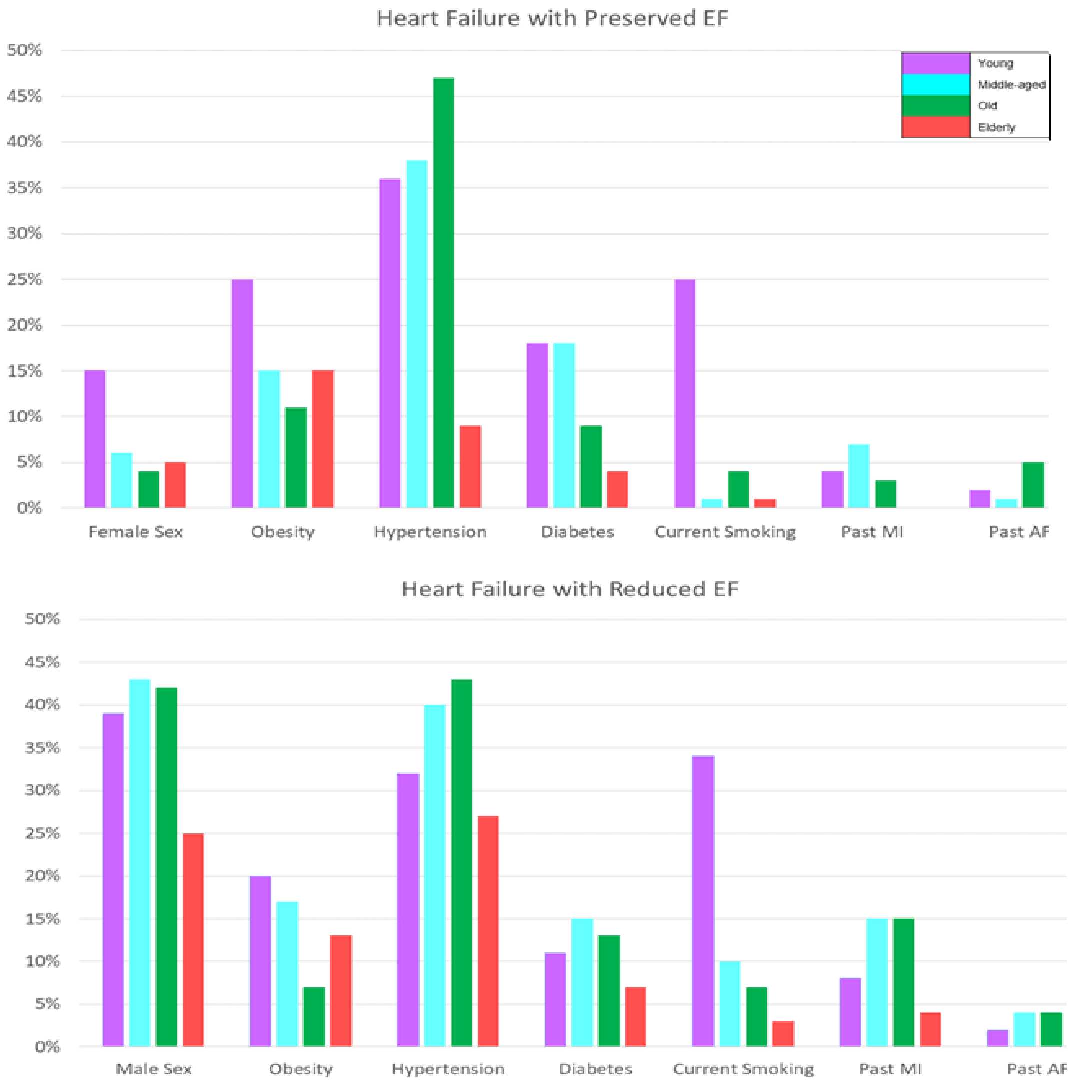

Supplemental methods on identification of heart failure cases in each cohort.

Framingham Heart Study (FHS).

The adjudication of heart failure cases in previous publications [1,2]. Briefly, an initial heart failure hospitalization was confirmed by a panel of three physicians after careful review of all available hospital and outpatient records using established protocols and criteria [2]. Major heart failure criteria include paroxysmal nocturnal dyspnea, elevated jugular venous pressure, rales, radiographic cardiomegaly, pulmonary edema, S3 gallop, and hepatojugular reflux, and minor criteria included bilateral ankle edema, nocturnal cough, dyspnea on exertion, hepatomegaly, pleural effusion, and tachycardia. Individuals had heart failure on the basis of two major or one major and two minor criteria. Given the wider availability of echocardiography after 1980, the cases occurring after 1980 were systematically classified as heart failure with reduced (HFrEF) and preserved (HFpEF) ejection fraction [1].

Prevention of Renal and Vascular End-stage Disease (PREVEND).

Identification of heart failure cases in PREVEND have been described previously [1,3]. In summary, heart failure events were adjudicated by review of hospital record based on the European Society of Cardiology Heart Failure Guidelines [4,5]. The diagnosis of HF included typical (breathlessness, orthopnea, paroxysmal nocturnal dyspnea, reduced exercise tolerance, fatigue, tiredness, ankle swelling) and atypical symptoms (nocturnal cough, wheezing, weight loss, bloated feeling, loss of appetite, confusion, depression, palpitations, syncope), and more specific (elevated jugular venous pressure, hepatojugular reflux, S3 gallop, laterally displaced left ventricular impulse, murmur) and less specific signs (edema, pulmonary crepitations, reduced air entry and dullness to percussion at lung bases, tachycardia, hepatomegaly, ascites, cachexia). An independent endpoint committee adjudicated all events by consensus [3].

Multi-Ethnic Study of Atherosclerosis (MESA).

Heart failure vents in the MESA cohort were independently adjudicated by two paired physicians, based on all available medical records, and study examination visits. Disagreements were classified by a full review committee. Only cases identified as definite HF were used in this study, and defined as symptoms of HF such as shortness of breath or edema, in addition to objective criteria including pulmonary edema or congestion by chest X ray, dilated ventricle or poor ventricular function, or left ventricular diastolic dysfunction [6].

References

1 Ho JE, Enserro D, Brouwers FP, *et al.* Predicting Heart Failure with Preserved and Reduced Ejection Fraction: The International Collaboration on Heart Failure Inftypes. *Circ Hear Fail* 2016;**9**:e003116. doi:10.1161/CIRCHEARTFAILURE.115.003116

2 Kannel WB, D’Agostino RB, Silbershatz H, *et al.* Profile for estimating risk of heart failure. *Arch Intern Med* 1999;**159**:1197–204. doi:10.1001/archinte.159.11.1197

3 Brouwers FP, De Boer RA, Van Der Harst P, *et al.* Incidence and epidemiology of new onset heart failure with preserved vs. reduced ejection fraction in a community-based cohort: 11-year follow-up of PREVEND. *Eur Heart J* 2013;**34**:1424–31. doi:10.1093/eurheartj/eht066

4 McMurray JJ V., Adamopoulos S, Anker SD, *et al.* ESC Guidelines for the diagnosis and treatment of acute and chronic heart failure 2012: The Task Force for the Diagnosis and Treatment

of Acute and Chronic Heart Failure 2012 of the European Society of Cardiology. Developed in collaboration with the Heart. *Eur Heart J* 2012;**33**:1787–847. doi:10.1093/eurheartj/ehs104

5 Paulus WJ, Tschöpe C, Sanderson JE, *et al.* How to diagnose diastolic heart failure: A consensus statement on the diagnosis of heart failure with normal left ventricular ejection fraction by the Heart Failure and Echocardiography Associations of the European Society of Cardiology. *Eur Heart J* 2007;**28**:2539–50. doi:10.1093/eurheartj/ehm037

6 Opdahl A, Ambale Venkatesh B, Fernandes VRS, *et al.* Resting heart rate as predictor for left ventricular dysfunction and heart failure: MESA (Multi-Ethnic Study of Atherosclerosis). *J Am Coll Cardiol* 2014;**63**:1182–9. doi:10.1016/j.jacc.2013.11.027
